# Lumbo-Costo-Vertebral Syndrome: An Iceberg with Tip of Hernia and Body of Spinal and Neurological Malformations

**DOI:** 10.21699/ajcr.v8i2.573

**Published:** 2017-03-18

**Authors:** Xenophon Sinopidis, Georgia Antoniou, Vasileios Alexopoulos, Antonios Panagidis, George Georgiou

**Affiliations:** 1 University of Patras, Greece; 2 Karamandaneion Children's Hospital, Patras, Greece

**Dear Sir**

We read with great interest the contribution of Gupta et al on the lumbo-costo-vertebral syndrome (LCVS).[1] We encountered this rare anomaly in a 39 week-of-gestation female patient, who after an uncomplicated birth underwent investigation because of a left lumbar hernia, and vertebral anomalies. Imaging assay with plain radiography, revealed the presence of tenth and eleventh thoracic hemivertebrae, and intervertebral cleft of the twelfth thoracic vertebra. These anomalies resulted in clockwise thoracic and counterclockwise lumbar scoliosis. Magnetic resonance imaging revealed syringomyelia at the level of the three lower thoracic dysplastic vertebrae, and low termination of the spinal cord at the level of the fourth lumbar vertebra.

Neurological examination at the neonatal age did not reveal any obvious deficit though reassessment at the age of fifteen months revealed impaired mobility of the lower limbs resulting in difficulty in walking for which assistance was needed. Inversion and adduction deformity of the left leg was present that resulted in mild scissor gait. A positive left Babinski sign was elicited but dorsiflexion of the foot was insufficient. On echocardiography patent foramen ovale found. Karyotyping did not reveal chromosome alterations. Kidney anatomy appeared normal on ultrasound. Surgical correction of the hernia, which contained small bowel and the lower pole of the spleen, was performed at the age of 18 months. Primary closure of the 3.5 cm diameter opening at the superior lumbar triangle was achieved without any further implications.

LCVS is considered as an uncommon type of congenital lumbar hernia which is present in all reported cases, combined with vertebral, genitourinary and rib anomalies.[1, 2] It has been considered as the fourth type of lumbar hernia after the superior lumbar triangle (Grynfeltt-Lesshaft triangle) hernia, inferior lumbar triangle (Petit triangle) hernia, and a diffuse anatomic type hernia.[2] Gupta et al presented a combination of a lumbar hernia with vertebral and rib dysmorphias.[1] In a case-series, tethered cord and diastematomyelia were described in half of the cases.[2] VACTERL anomaly has been reported in combination with LCVS.[3] LCVS with syringomyelia, posterior spinal dysraphism, and lipomas adherent through a spina bifida to the dorsal surface of a tethered spinal cord was treated with lumbar exploration and laminectomy in one report.[4]

There are no treatment guidelines are available as handful of cases are reported.[5] The lumbar hernia is the prominent component of the anomaly. A meticulous follow-up is mandatory for timely therapeutic interventions for hidden anomalies to prevent neurologic morbidity.

## Footnotes

**Source of Support:** Nil

**Conflict of Interest:** None declared

